# The relationship between discharge destination and rehospitalization in Chinese patients with heart failure: a cohort study

**DOI:** 10.3389/fcvm.2026.1741085

**Published:** 2026-04-23

**Authors:** Xia Yun, Shaojie Bi, Xiaohua Shi, Wen An, Jie Wen, Hui Wu, Chunyan Wang, Zhen Liu

**Affiliations:** 1 Department of Geriatrics, The Second Qilu Hospital of Shandong University, Jinan, China; 2 Department of Cardiology, The Second Qilu Hospital of Shandong University, Jinan, China

**Keywords:** China, discharge destination, heart failure, hospitalized patients, rehospitalization

## Abstract

**Background:**

The high readmission rate among heart failure (HF) patients is a significant concern, but limited research has explored the relationship between discharge destination and readmission rates in Chinese HF patients.

**Objective:**

To examine the association between discharge destination and readmission in HF patients.

**Methods:**

This retrospective cohort study analyzed data from 2,008 HF patients admitted to the Fourth People's Hospital of Zigong City, China, between December 2016 and June 2019. Patients were divided into two groups based on their discharge destination: home and healthcare facility. The primary outcome was readmission within 6 months, with secondary outcomes at 3 months and 28 days. Cox proportional hazards regression and Kaplan–Meier curves were used to assess the relationship, with additional subgroup and interaction analyses for robustness.

**Results:**

A total of 836 patients with HF were enrolled in this study. Among them, 638 patients were discharged to home, while 198 patients were discharged to healthcare facilities. The multivariable Cox proportional hazards regression model revealed that discharge to healthcare facilities were independently associated with a higher readmission risk compared with discharge to home, adjusted HR 1.26, 95% CI 1.06–1.50, *P* = 0.009 for readmission within 6 months. Similar trends were observed for readmission within 3 months (HR: 1.26, 95% CI: 1.02–1.55, *P* = 0.034) and 28 days (HR: 1.53, 95% CI: 1.04–2.26, *P* = 0.032). Kaplan–Meier analysis (log-rank test *P* = 0.011) supported the above findings, and the association remained robust in subgroup and sensitivity analyses.

**Conclusion:**

This study demonstrated that discharge to a healthcare facility was independently associated with an increased readmission risk among HF patients. Discharge destination should be considered as a potential indicator of readmission risk in clinical practice.

## 1 Introduction

Heart failure (HF) represents the terminal stage of heart disease and is frequently described as the “last battlefield” in the domain of cardiovascular disorders (1). The high incidence, readmission rates, mortality rates, and associated medical costs linked to HF pose significant challenges for healthcare systems globally.

Research indicates that over 20% of patients experience readmission within 30 days following their initial hospitalization for HF, with the readmission rate within six months reaching up to 50% (2, 3).

Furthermore, nearly 80% of the treatment costs for HF are attributable to hospitalization and short-term readmissions (4). Consequently, accurately assessing the risk of short-term readmission and implementing targeted interventions have become crucial for mitigating readmission rates and alleviating the socioeconomic burden associated with this condition (5).

For the majority of patients, the primary objective following discharge is to return home. However, over 40% of patients require post-acute care after hospitalization (6), and among those receiving such care, up to 30% may necessitate readmission for further treatment (7), Discharge destinations for HF patients are generally categorized as home discharge or healthcare facility (8). In China, healthcare facility refers to institutions capable of providing medical intervention or professional nursing care post-discharge, primarily including transfers to other hospitals, community hospitals, and convalescent or nursing homes (9).

Previous studies have explored the relationship between discharge destination and clinical outcomes, with some investigating discharge destination as a primary outcome in various surgical domains, including vascular, orthopedic, and abdominal surgeries (10–12). Notably, research suggests that HF patients who are acutely admitted and subsequently transferred to rehabilitation or long-term care facilities exhibit significantly lower one-year readmission rates compared to those discharged home (8). However, conflicting evidence exists, with certain studies indicating that the prognosis for HF patients discharged to non-home destinations may be inferior to that of those discharged home (13). The conclusions drawn from studies conducted across different countries and populations vary, underscoring the need for further research on this association in the Chinese HF population. Therefore, this study aims to evaluate the discharge destinations (home and healthcare facility) of HF patients in China and analyze the relationship between these destinations and readmission rates.

## 2 Materials and methods

### 2.1 Setting

This retrospective cohort study utilized data from a publicly accessible Chinese population database hosted on PhysioNet (https://physionet.org/content/heart-failure-zigong/1.3/) (14, 15). Prior to data acquisition, the corresponding author (Dong) completed the requisite training program administered by the National Institutes of Health, thereby obtaining authorized access to the database (Certification ID: 58380637). The dataset gathered information on a total of 2,008 adult HF patients from December 2016 to June 2019 at the Fourth People's Hospital in Zigong, Sichuan, China, to comprehend the characteristics of the Chinese HF population (15, 16). The dataset includes demographic information, baseline clinical characteristics, co-morbidities, laboratory test results, and outcomes. The reporting of this study adhered to the STROBE (Strengthening the Reporting of Observational Studies in Epidemiology) statement.

This study was approved by the Ethics Committee of the Fourth People's Hospital of Zigong (approval number: 2020-010). The requirement for informed consent was waived due to the retrospective design of this study. The study was conducted in strict accordance with the Declaration of Helsinki (17).

### 2.2 Study population

This study enrolled patients diagnosed with HF from the database, which included 2,008 patients. The diagnosis of HF is established according to the European Society of Cardiology (ESC) diagnostic criteria (18): Specifically, patients were required to present with typical symptoms and/or signs of HF (e.g., breathlessness, fatigue, peripheral edema, elevated jugular venous pressure), supported by objective evidence of cardiac structural or functional abnormalities (e.g., reduced left ventricular ejection fraction, echocardiographic abnormalities), and/or elevated natriuretic peptide levels (BNP > 35 pg/mL or NT-proBNP > 125 pg/mL), as detailed in the original database documentation. Patients with a diagnosis of HF at hospital admission, as recorded using ICD-9 codes in the electronic health record, were eligible for inclusion (5).

The exclusion criteria were as follows: (1) missing discharge status (unknown or died); (2) missing readmission time data. Consequently, a total of 836 patients were ultimately included in the analysis.

### 2.3 Study variables

We obtained discharge disposition information from medical records, classifying it into two types: home group or healthcare facility group. The primary outcome was defined as rehospitalization within 6 months, while secondary outcomes included rehospitalization within 3 months and within 28 days. Rehospitalization was defined as all-cause rehospitalization within the specified timeframes (28 days, 3 months, and 6 months), indicating any readmission regardless of cause. If patients were unable to reach the clinical center, the follow-up visit was replaced by a telephone call (15). Due to the nature of the database, we were unable to distinguish readmissions by etiology or whether they were planned events.

The data included demographic information, baseline clinical characteristics, comorbidities, and laboratory findings, all of which were obtained on the day of hospital admission. Only the first admission for a patient was included in the cohort if they were subsequently readmitted.

### 2.4 Statistical analysis

Categorical data were expressed as percentages, whereas normally distributed continuous data were reported as means ± standard deviations (SD), and non-normally distributed variables were described using medians and interquartile ranges (IQR). Hazard ratios (HR) were estimated using Cox proportional hazards models, adjusted for potential confounding factors.

In the multivariable analysis, we employed various statistical models to assess the stability of the results. We adjusted the factors based on the following three criteria: (1) Variables were included if their addition to the model resulted in a change of at least 10% in the matched odds ratio. (2) In the univariate analysis, we adjusted for variables with *p*-values less than 0.1 (Supplementary Tables 1–3). (3) In the multivariable analysis, variables were selected based on prior findings and clinical considerations.

The cumulative 6-month incidence of the outcome measures was estimated using the Kaplan–Meier method, and differences were evaluated using the log-rank test. Model covariates were selected *a priori* based on prior prognostic reports concerning HF patients and clinical expertise. The covariates included age, sex, body mass index(BMI), occupation status, admission way, diabetes, New York Heart Association (NYHA) classification, Killip grade, type of HF, Charlson Comorbidity Index (CCI) score, chronic obstructive pulmonary disease (COPD), white blood cell count, platelet count, creatinine (Cre), D-dimer, high-sensitivity cardiac troponin (hs-cTn), N-terminal pro-B-type natriuretic peptide (NT-proBNP), albumin(ALB), liver disease, Glomerular Filtration Rate (GFR), discharge day.

We employed a robust statistical approach to manage missing data through multiple imputation with five imputations using the chained equations method within the R ‘mice’ package. Subgroup analyses were conducted using stratified Cox proportional hazards models, and interactions among subgroups were assessed using the likelihood ratio test. To address the potential heterogeneity introduced by including patients whose primary admission diagnosis might not have been HF, we conducted a sensitivity analysis. This involved excluding all patients with major comorbidities—including chronic obstructive pulmonary disease, chronic kidney disease, diabetes mellitus, liver disease, cerebrovascular disease, peptic ulcer disease, peripheral vascular disease, malignant tumors, and acquired immunodeficiency syndrome (AIDS)—from the original cohort. Ultimately, 302 patients with HF as the sole primary diagnosis were included in the analysis (Supplementary Table 4). A Multivariable Cox proportional hazards model was subsequently applied to this refined cohort. All analyses were conducted using the statistical software packages R version 3.3.2 (http://www.R-project.org, The R Foundation) and Free Statistics software version 2.0. Two-sided *p*-values < 0.05 were regarded as statistically significant.

## 3 Results

### 3.1 Baseline clinical characteristics

**Figure 1:**
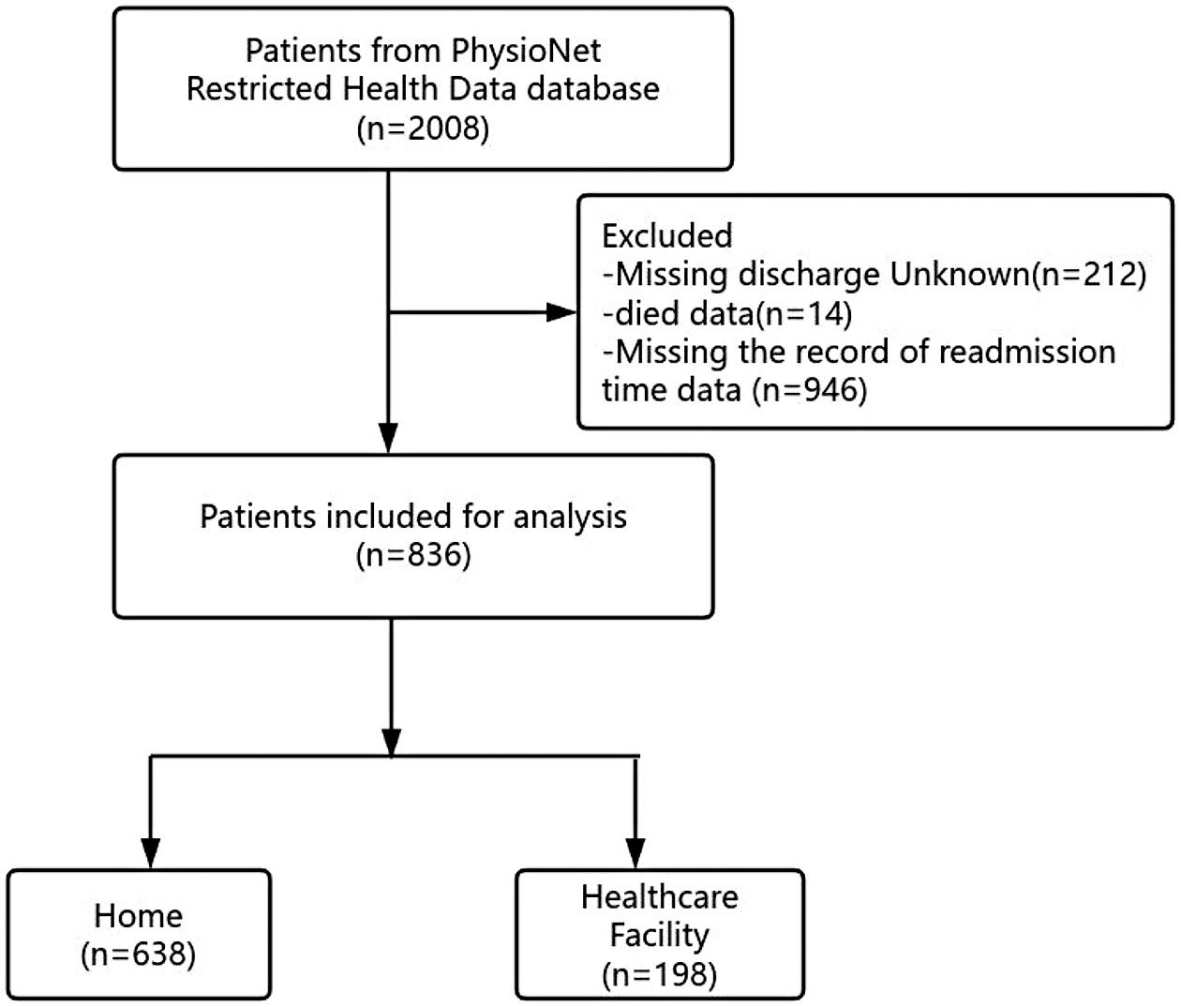
Flowchart of patient selection.

In the original database, there were 2008 patients with HF. After excluding patients with missing records of readmission time data and missing discharge status (Unknown or died), a total of 836 patients were included in this study for analysis. Among the enrolled 836 patients, 638(76.3%)were discharged to home and 198 (23.7%)were discharged to a healthcare facility (Figure 1).

Table 1 presents the demographic, comorbidity, and baseline characteristics of HF patients discharged to home (638 patients) and those discharged to healthcare facilities (198 patients). Among the total of 836 patients, 92.0% were aged 60 years or older, and 43.2% were male. The majority of patients identified as urban residents, comprising 88.6% of the study population. Notably, the proportion of patients admitted through the emergency department was significantly higher in the healthcare facility group, at 58.1%, compared to the home discharge group.

**Table 1:** Baseline characteristics of the participants.

Variables	Total (*n* = 836)	Home (*n* = 638)	Healthcare facility (*n* = 198)	*P*
Sex, male, *n* (%)	361 (43.2)	278 (43.6)	83 (41.9)	0.681
Age (years), *n* (%)				0.488
<60	67 (8.0)	49 (7.7)	18 (9.1)	
≥60 and <80	465 (55.6)	362 (56.7)	103 (52)	
≥80	304 (36.4)	227 (35.6)	77 (38.9)	
Occupation, *n* (%)				0.001
Farmer	51 (6.2)	46 (7.3)	5 (2.6)	
Urban Resident	727 (88.6)	540 (86.3)	187 (95.9)	
Worker/Others	43 (5.2)	40 (6.4)	3 (1.5)	
Admission way, *n* (%)				< 0.001
Non Emergency	453 (54.2)	370 (58)	83 (41.9)	
Emergency	383 (45.8)	268 (42)	115 (58.1)	
BMI (kg/m^2^)	21.7 ± 13.8	21.2 ± 4.0	23.3 ± 27.4	0.066
NYHA *n* (%)				0.238
II	108 (12.9)	89 (13.9)	19 (9.6)	
III	436 (52.2)	332 (52)	104 (52.5)	
IV	292 (34.9)	217 (34)	75 (37.9)	
Killip grade, *n* (%)				0.427
I	229 (27.4)	177 (27.7)	52 (26.3)	
II	433 (51.8)	335 (52.5)	98 (49.5)	
III	149 (17.8)	106 (16.6)	43 (21.7)	
IV	25 (3.0)	20 (3.1)	5 (2.5)	
Comorbidities				
COPD, *n* (%)	96 (11.5)	76 (11.9)	20 (10.1)	0.485
Diabetes, *n* (%)	230 (27.5)	177 (27.7)	53 (26.8)	0.788
CKD, *n* (%)	232 (27.8)	173 (27.1)	59 (29.8)	0.462
Liver disease, *n* (%)	33 (4.0)	25 (3.9)	8 (4.1)	0.929
Type II respiratory failure, *n* (%)	39 (4.7)	32 (5)	7 (3.5)	0.388
Laboratory values				
GFR (mL/min/1.73m^2^)	65.5 ± 36.4	65.7 ± 37.5	64.9 ± 32.6	0.798
Cre (umol/L)	93.0 (68.5, 129.1)	93.8 (68.7, 132.2)	92.6 (68.5, 123.7)	0.611
White blood cell (10^9/L)	7.1 ± 3.3	7.2 ± 3.4	6.9 ± 3.2	0.312
Red blood cell (10^9/L)	3.8 ± 0.8	3.8 ± 0.8	3.8 ± 0.7	0.495
Platelet (10^9/L)	141.5 ± 65.0	138.4 ± 58.5	151.5 ± 82.3	0.014
K (mmol/L)	4.0 ± 0.7	4.0 ± 0.7	4.0 ± 0.7	0.601
Na (mmol/L)	137.9 ± 4.8	138.0 ± 4.7	137.8 ± 5.1	0.656
D-dimer (mg/L)	1.1 (0.8, 2.0)	1.2 (0.8, 2.1)	1.1 (0.7, 1.6)	0.03
Hs-cTn (ng/mL)	0.1 (0.0, 0.1)	0.1 (0.0, 0.1)	0.1 (0.0, 0.2)	0.475
AST (IU/L)	26.0 (19.0, 37.0)	26.0 (19.0, 36.0)	26.0 (20.0, 38.0)	0.377
CK-MB (IU/L)	15.9 (12.0, 21.5)	15.9 (12.1, 21.6)	15.2 (11.5, 21.3)	0.383
NT-proBNP (pg/mL)	804.7 (299.3, 1,789.0)	791.6 (308.8, 1,738.5)	810.0 (277.5, 1,921.0)	0.991
ALB (g/L)	36.9 ± 4.8	36.9 ± 5.0	36.8 ± 4.4	0.797
Scoring system				
CCI Score	2.0 ± 1.0	2.0 ± 1.0	2.0 ± 1.1	0.645
GCS	14.9 ± 0.7	14.9 ± 0.8	14.9 ± 0.6	0.913
Movement	6.0 ± 0.4	6.0 ± 0.5	6.0 ± 0.2	0.507
Discharge Day (d)	10.4 ± 9.1	10.6 ± 10.1	9.7 ± 4.9	0.203
Outcome				
Readmission within 28-d, *n* (%)	127 (15.2)	87 (13.6)	40 (20.2)	0.025
Readmission within 3 m, *n* (%)	461 (55.1)	339 (53.1)	122 (61.6)	0.036
Readmission within 6 m, *n* (%)	712 (85.2)	533 (83.5)	179 (90.4)	0.018

Categorical data are presented as percentages, continuous data are presented as median and interquartile range (IQR). BMI, body mass index; NYHA, New York Heart Association; COPD, chronic obstructive pulmonary disease; CKD, chronic kidney disease; CCI, Charlson comorbidity index; GFR, glomerular filtration rate; CK-MB, creatine kinase isoenzyme; NT-proBNP N-terminal pro-B-type natriuretic peptide; Hs-cTn, high-sensitivity cardiac troponin; K, potassium ion; ALB, albumin; AST, glutamic oxaloacetic transaminase; GCS, Glasgow coma scale, CI, 95% conﬁdence intervals.

Additionally, significant differences were observed between the two groups in terms of platelet counts and D-dimer levels. Over time, readmission rates for HF patients increased, with the healthcare facility group exhibiting higher rates of readmission at 28 days, 3 months, and 6 months. Importantly, there were no significant differences between the two groups regarding gender, age, body mass index, NYHA classification, or comorbidities, as indicated by *P*-values greater than 0.05.

### 3.2 Association between discharge destination and clinical outcomes

The multivariable regression results (Table 2), demonstrate a significant association between discharge location and hospital readmission rates. For patients discharged to healthcare facilities, the hazard ratio (HR) for readmission within 6 months was 1.26 (95% CI: 1.06–1.50, *P* = 0.009) in Model 3, indicating a notable increase in risk compared to those discharged home. Similarly, readmission within 3 months showed an HR of 1.26 (95% CI: 1.02–1.55, *P* = 0.034), and for readmissions within 28 days, the HR was 1.53 (95% CI: 1.04–2.26, *P* = 0.032). These results consistently highlighted the elevated risk of readmission associated with healthcare facility discharge across various follow-up periods.

**Table 2:** Association between discharge destination and readmission in multiple regression model.

Variable		Unadjusted model		Model1		Model2		Model3	
		HR (95% CI)	*P* value	HR (95% CI)	*P* value	HR (95%CI)	*P* value	HR (95% CI)	*P* value
Readmission within 28-d	Home	1(Ref)		1(Ref)		1(Ref)		1(Ref)	
	Healthcare facility	1.54 (1.06∼2.23)	0.025	1.54 (1.05∼2.25)	0.026	1.52 (1.04∼2.23)	0.031	1.53 (1.04∼2.26)	0.032
Readmission within 3m	Home	1(Ref)		1(Ref)		1(Ref)		1(Ref)	
	Healthcare facility	1.24 (1.01∼1.52)	0.042	1.29 (1.05∼1.6)	0.017	1.26 (1.02∼1.56)	0.031	1.26 (1.02∼1.55)	0.034
Readmission within 6m	Home	1(Ref)		1(Ref)		1(Ref)		1(Ref)	
	Healthcare facility	1.25 (1.05∼1.48)	0.011	1.29 (1.08∼1.53)	0.005	1.28 (1.07∼1.53)	0.006	1.26 (1.06∼1.5)	0.009

Model 1: was adjusted for sex, age, BMI; Model 2:was adjusted for the variables in model1 + NYHA, Killip grade, type of HF, CCI score; Model 3: was adjusted for the variables in model 2 + COPD, diabetes, white blood cell, platelet, D-dimer, Hs-cTn, NT-pro BNP, ALB, liver disease, GFR, discharge day, occupation status and admission way.

**Figure 2:**
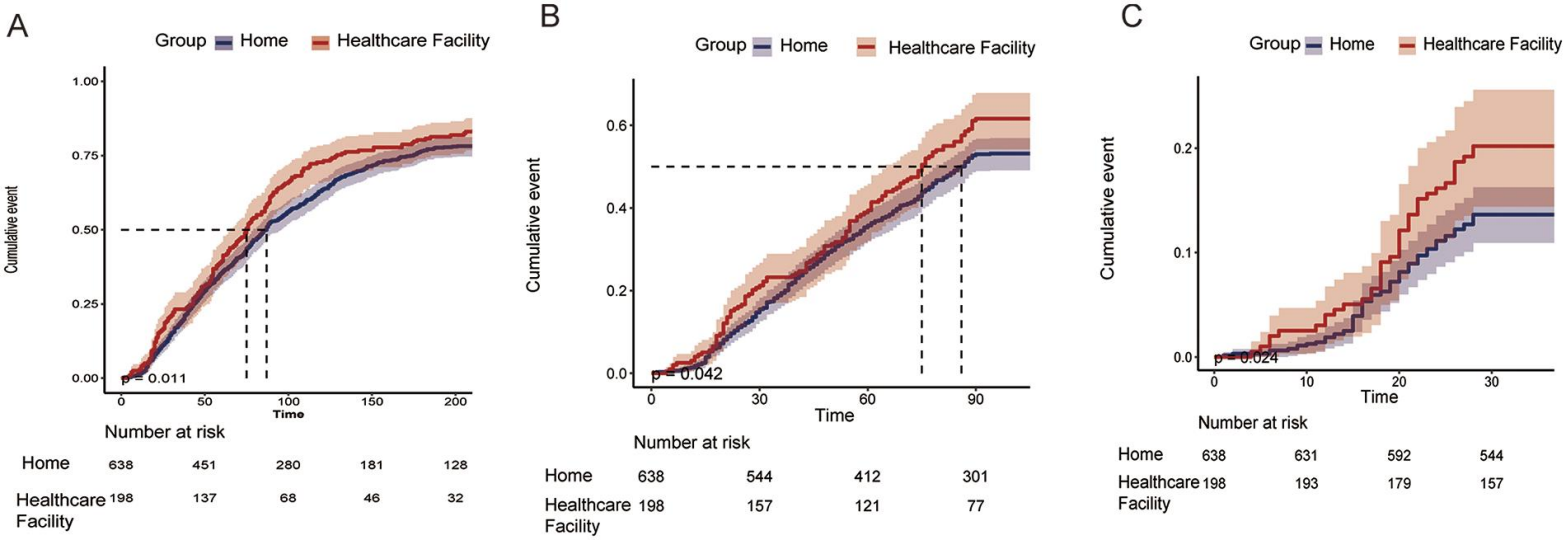
Cumulative incidence of readmission in HF patients. **(A)**: 6-month readmission **(B)**: 3-month readmission **(C)**: 28-day readmission.

In addition, the cumulative readmission rate at 6 months was significantly higher in the healthcare facility group. This conclusion was confirmed by Kaplan–Meier curve analysis (*P* = 0.011, Figure 2A). Similar patterns were observed in Kaplan–Meier curve analysis for readmission within 3 months and 28 days (*P* < 0.005, Figures 2B, C).

### 3.3 The results of sensitivity analyses

**Figure 3:**
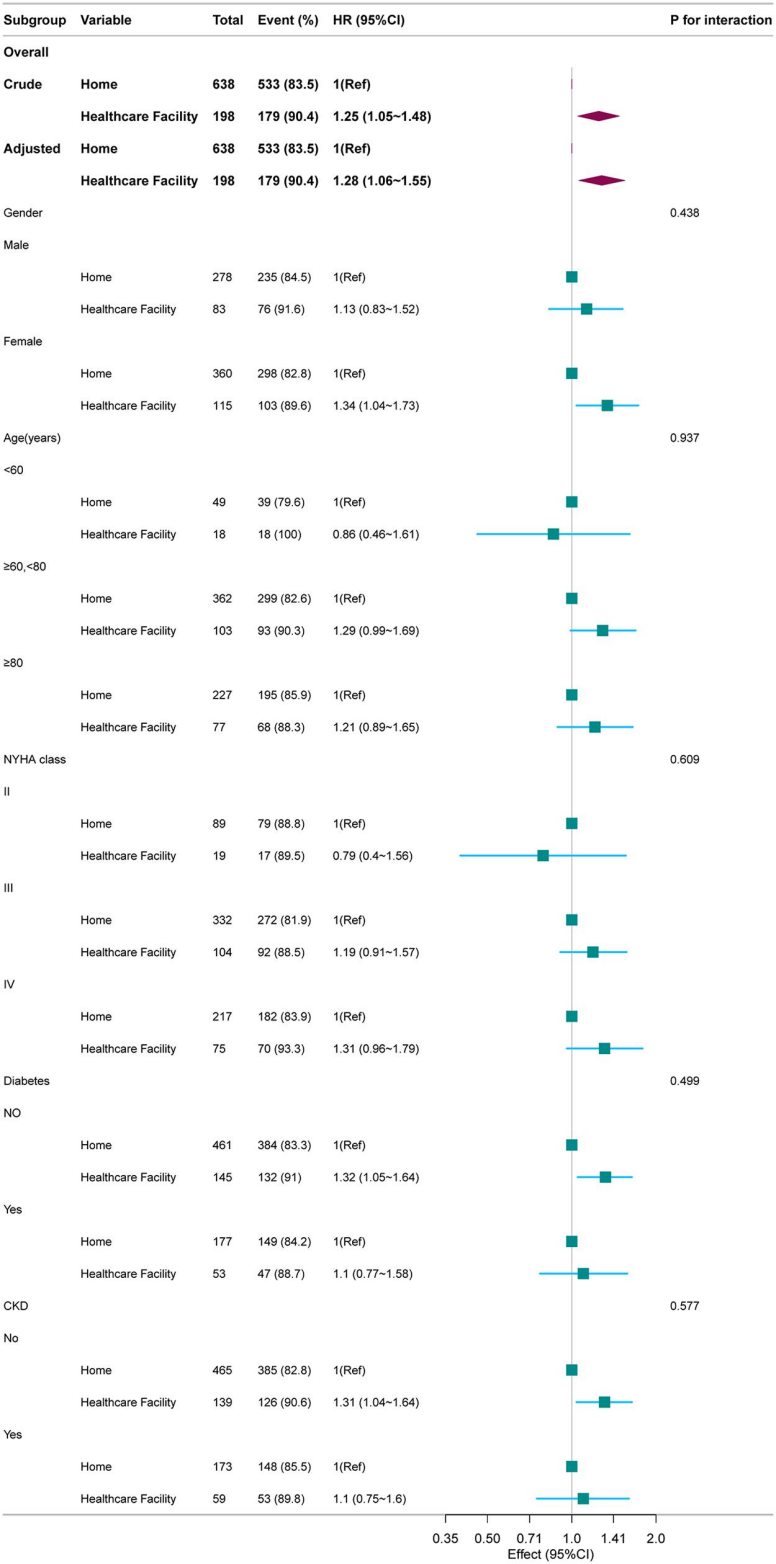
Association between discharge destination and the hazard ratio of readmission within 6 months according to subgroup. The stratifications were adjusted for all variables (Killip grade, type of HF, CCI score, COPD, diabetes, white blood cell, platelet, cre, D-dimer, Hs-cTn, NT-proBNP, liver disease, Occupation status and Admission way) except for the stratification factor itself. Squares represent the HRs and horizontal lines represent 95% CIs. Diamonds represent the overall HR, and the outer points of the diamonds represent the 95% CI.

The results of the stratified analysis showed that after adjusting for confounding factors, subgroup analyses stratified by age, sex, NYHA functional class, diabetes, and CKD revealed a generally consistent trend in the effect of discharge destination on readmission risk among patients with HF across all subgroups. No significant interaction was observed (*P* for interaction > 0.05) (Figure 3). Similar results were observed in the stratified analysis for readmission within 28 days and 3 months (see Supplementary Figures 1, 2). In the cohort analysis excluding HF patients with other comorbidities, the Multivariate Cox proportional hazards model demonstrated that the association between discharge destination and HF readmission risk remained consistent with the primary analysis (see Supplementary Table 5).

## 4 Discussion

In this retrospective cohort study of Chinese patients with HF, we identified a significant association between discharge destination and hospital readmission risk—an association that remained robust after adjusting for multiple confounding factors. Specifically, patients discharged to healthcare facilities had a higher incidence of emergency admissions (58.1%) at baseline, with 88.6% of the overall cohort being urban residents. Notably, readmission rates for HF increased progressively over time, with those discharged to healthcare facilities experiencing significantly higher risks at 28 days, 3 months, and 6 months post-discharge. These findings were consistent across clinical subgroups and validated by sensitivity analyses, aligning with research by Madeline R et al. (19) in the United States, which reported elevated readmission and 30-day mortality risks in HF patients discharged to non-home settings, persisting for up to 90 days.

Currently, there is no uniform definition for the timing of readmission: 30 days (20), 90 days (21, 22), and 180 days (23). have all been proposed as relevant time windows. To address this, we adopted three post-discharge time points—28 days, 3 months, and 6 months—to capture distinct clinical phases: the early phase (∼4 weeks, approximating the conventional 30-day window) reflects in-hospital care quality and care transitions (24); the subacute phase (∼3 months) indicates incomplete recovery or early disease progression (25); and the mid-term phase (6 months) provides insights into disease stability, treatment adherence, and late complications (26). This stratified approach not only clarifies time-dependent readmission patterns but also facilitates the identification of phase-specific predictors (27).

The proportion of patients discharged to home varies considerably across countries. Our cohort showed 76.3% home discharge vs. 23.7% facility transfer, contrasting with Canadian (64.8% home) (28), and Japanese (87% home) (13) data. These differences likely reflect variations in geographic environments and national healthcare systems. In practice, discharge destination is influenced by a combination of factors—including social background, economic status, and functional capacity—rather than being determined solely by medical stability at the time of discharge.

Existing evidence on the impact of discharge destination on HF outcomes remains inconsistent. Washida et al. (13) linked non-home discharge to functional decline, reduced self-care ability, worsening HF, prolonged hospital stays, and poorer prognoses, while Wang et al. (9) observed an association between non-home discharge and increased mid-term mortality in elderly patients with acute myocardial infarction. In contrast, Japanese (8) and Masip et al. (29) studies reported lower readmission rates in patients discharged to healthcare facilities, attributed to structured disease management (e.g., medication oversight, lifestyle interventions, and physical rehabilitation). These discrepancies may stem from variations in study populations, HF definitions, and clinical practice patterns across regions.

In our cohort, most patients discharged to healthcare facilities were admitted via emergency services, indicating more severe acute conditions or poorer functional status that necessitated post-acute care. A Japanese multicenter study (8) similarly found that HF patients transferred to rehabilitation or long-term care facilities were older, with higher inflammation, poorer nutritional status, and lower daily living capabilities. Notably, even after Multivariate adjustment, patients discharged to healthcare facilities had a higher readmission risk. To further validate this finding, we performed a sensitivity analysis restricted to patients with HF as the sole diagnosis to exclude comorbidity-related heterogeneity. The results remained consistent with the primary analysis, indicating that the association between discharge destination and readmission risk is robust.

Several mechanisms may explain the higher readmission risk in patients discharged to healthcare facilities. These patients often have more severe illness and functional impairment (8, 30), requiring additional rehabilitation and nursing support. Non-home discharge may also mean reduced family emotional support, exacerbating depression and anxiety—factors known to worsen HF outcomes (30–32), as positive psychological status is critical for prolonging HF remission and reducing readmissions. Furthermore, closer monitoring by nursing staff in healthcare facilities enables timely identification of high-risk individuals and prompt referral for inpatient treatment, which may partly explain the higher readmission rate in this group. Collectively, these findings highlight discharge destination as a convenient, readily available marker for readmission risk, which could be integrated into HF risk stratification tools to prioritize enhanced follow-up (e.g., weekly telephone check-ins, monthly outpatient visits) for high-risk patients. Strengthening collaboration between cardiology departments, healthcare facilities, community services, and home-based care—along with standardized disease management pathways and individualized care plans—are crucial strategies to reduce readmissions (33). As supported by evidence that multidisciplinary transitional care effectively mitigates HF readmission risk (34, 35).

Several limitations should be acknowledged. First, as an observational study, it cannot establish causality between discharge destination and readmission risk. Second, the use of a single-center database limits generalizability to other populations. Third, the database did not provide detailed information on psychological or socioeconomic factors, such as medication adherence, reasons for readmission, or family support. Fourth, although we adjusted for a wide range of clinical covariates and conducted a sensitivity analysis excluding patients with major comorbidities to minimize confounding, unmeasured selection bias and residual confounding—such as frailty or functional status at admission—may still exist. Despite these limitations, this study effectively elucidates the relationship between discharge destination and readmission in patients with HF, providing valuable evidence in this field.

## 5 Conclusions

This study demonstrates that discharge to a healthcare facility correlates with an elevated readmission rate among HF patients, suggesting that the discharge destination could serve as a reliable predictor of readmission risk for this population. These findings offer critical insights for optimizing care pathways and developing targeted strategies to mitigate readmission rates.

## Data Availability

This retrospective cohort study utilized data from a publicly accessible Chinese population database hosted on PhysioNet (https://physionet.org/content/heart-failure-zigong/1.3/).
