# Supplementary material for: The relationship between discharge destination and rehospitalization in Chinese patients with heart failure: a cohort study
Source: Front Cardiovasc Med. 2026 Apr 23;13:1741085. doi: 10.3389/fcvm.2026.1741085 (PMC13149289; doi:10.3389/fcvm.2026.1741085)
Supplement: Supplementary file 1 [file Datasheet1.docx]

**Supplementary Table 1 Univariate Cox regression analysis of risk factors associated with readmission in heart failure within 6 month**

| Variable | HR(95%CI) | *P* value |
| --- | --- | --- |
| Healthcare facility | 1.25 (1.05,1.48) | 0.011 |
| Emergency admission way | 1.04 (0.89,1.2) | 0.644 |
| Age＞80(years) | 1.22 (0.98,1.52) | 0.077 |
| Male | 0.93 (0.8,1.08) | 0.346 |
| BMI (kg/m^2^) | 1.0036 (0.9971,1.0101) | 0.276 |
| NYHA class IV | 0.99 (0.78,1.25) | 0.929 |
| Killip grade IV | 1.74 (1.14,2.66) | 0.011 |
| Dementia | 0.92 (0.69,1.22) | 0.548 |
| Diabetes | 1.08 (0.91,1.27) | 0.375 |
| CKD | 1.12 (0.95,1.32) | 0.169 |
| liver.disease | 1.28 (0.88,1.87) | 0.202 |
| movement | 0.96 (0.81,1.15) | 0.668 |
| Discharge Day | 1.0084 (1.0018,1.015) | 0.012 |
| CCI | 1.06 (0.98,1.14) | 0.133 |
| GCS | 0.97 (0.87,1.08) | 0.536 |
| Cre (mmol/l) | 1.0017 (1.0008,1.0025) | < 0.001 |
| White blood cell (10^9/L) | 1.0031 (0.9813,1.0253) | 0.783 |
| Red blood cell (10^9/L) | 0.92 (0.84,1.02) | 0.112 |
| Platelet (10^9/L) | 1.0005 (0.9993,1.0016) | 0.436 |
| D-dimer (mg/L) | 0.9976 (0.9754,1.0202) | 0.831 |
| Hs-cTn (ng/mL) | 1.14 (1.01,1.29) | 0.039 |
| AST ( IU/L ) | 1 (0.9995,1.0005) | 0.943 |
| NT-proBNP(pg/ml ) | 1.0001 (1.0001,1.0002) | < 0.001 |
| CRP (mg/L) | 1.0001 (0.9968,1.0034) | 0.965 |
| ALB (g/L) | 0.98 (0.97,1) | 0.05 |

Abbreviations: BMI, body mass index; NYHA, New York heart association; COPD,Chronic obstructive pulmonary disease,CKD chronic kidney disease,CCI, Charlson Comorbidity Index; GFR glomerular filtration rate,CK-MB Creatine kinase isoenzyme,NT-proBNP N-terminal pro-B-type natriuretic peptide,Hs-cTn,high-sensitivity cardiac troponin,K Potassium ion,ALB Albumin,AST glutamic oxaloacetic transaminase, GCS,Glasgow Coma Scale.

**Supplementary Table 2 Univariate Cox regression analysis of risk factors associated with readmission in heart failure within 3 month**

| Variable | HR(95%CI) | *P* value |
| --- | --- | --- |
| Healthcare facility | 1.24 (1.01,1.52) | 0.042 |
| Emergency admission way | 1.11 (0.92,1.33) | 0.278 |
| Age＞80(years) | 1.33 (1,1.77) | 0.05 |
| Male | 0.98 (0.81,1.18) | 0.811 |
| BMI(kg/m^2^) | 1.0042 (0.9983,1.0101) | 0.166 |
| NYHA class IV | 1.18 (0.87,1.59) | 0.29 |
| Killip grade IV | 1.8 (1.1,2.96) | 0.02 |
| Dementia | 0.76 (0.52,1.13) | 0.177 |
| Diabetes | 1.11 (0.91,1.36) | 0.297 |
| CKD | 1.15 (0.94,1.4) | 0.184 |
| Liver disease | 1.54 (1.01,2.34) | 0.045 |
| movement | 0.95 (0.77,1.16) | 0.594 |
| Discharge Day | 1.0072 (0.9995,1.015) | 0.068 |
| CCI | 1.09 (0.99,1.19) | 0.071 |
| GCS | 0.94 (0.83,1.06) | 0.307 |
| Cre (mmol/l) | 1.0014 (1.0005,1.0024) | 0.004 |
| White blood cell (10^9/L) | 1.01 (0.98,1.04) | 0.462 |
| Red blood cell (10^9/L) | 0.9 (0.8,1.02) | 0.09 |
| Platelet (10^9/L) | 1.0006 (0.9992,1.002) | 0.391 |
| D-dimer (mg/L) | 0.9966 (0.9711,1.0228) | 0.798 |
| Hs-cTn (ng/mL) | 1.28 (1.14,1.44) | < 0.001 |
| AST ( IU/L ) | 1.0003 (0.9998,1.0009) | 0.247 |
| NT-proBNP(pg/ml ) | 1.0001 (1,1.0002) | < 0.001 |
| CRP (mg/L) | 0.9998 (0.9957,1.0039) | 0.923 |
| ALB (g/L) | 0.98 (0.96,1) | 0.043 |

Abbreviations: BMI, body mass index; NYHA, New York heart association; COPD,Chronic obstructive pulmonary disease,CKD chronic kidney disease,CCI, Charlson Comorbidity Index; GFR glomerular filtration rate,CK-MB Creatine kinase isoenzyme,NT-proBNP N-terminal pro-B-type natriuretic peptide,Hs-cTn,high-sensitivity cardiac troponin,K Potassium ion,ALB Albumin,AST glutamic oxaloacetic transaminase, GCS,Glasgow Coma Scale.

**Supplementary Table 3 Univariate Cox regression analysis of risk factors associated with readmission in heart failure within 28-day**

| Variable | HR(95%CI) | *P* value |
| --- | --- | --- |
| Healthcare facility | 1.54 (1.06,2.23) | 0.025 |
| Emergency admission way | 1.23 (0.87,1.74) | 0.251 |
| Age＞80(years) | 0.91 (0.56,1.46) | 0.687 |
| Male | 0.83 (0.58,1.17) | 0.279 |
| BMI(kg/m^2^) | 1.005 (0.9998,1.0103) | 0.058 |
| NYHA class IV | 1.61 (0.88,2.95) | 0.123 |
| Killip grade IV | 2.61 (1.06,6.44) | 0.037 |
| Dementia | 0.65 (0.28,1.46) | 0.295 |
| Diabetes | 1.43 (0.99,2.06) | 0.056 |
| CKD | 1.3 (0.9,1.88) | 0.163 |
| liver.disease | 1.7 (0.83,3.48) | 0.146 |
| movement | 1.0068 (0.6391,1.5862) | 0.977 |
| Discharge Day | 0.97 (0.95,1) | 0.096 |
| CCI | 1.08 (0.91,1.28) | 0.356 |
| GCS | 0.95 (0.76,1.19) | 0.667 |
| Cre (mmol/l) | 1.0014 (0.9998,1.003) | 0.091 |
| White blood cell (10^9/L) | 1.03 (0.99,1.08) | 0.143 |
| Red blood cell (10^9/L) | 0.91 (0.72,1.15) | 0.435 |
| Platelet (10^9/L) | 1.0003 (0.9977,1.003) | 0.812 |
| D-dimer (mg/L) | 0.98 (0.92,1.05) | 0.614 |
| Hs-cTn (ng/mL) | 1.36 (1.2,1.53) | < 0.001 |
| AST ( IU/L ) | 1.001 (1.0004,1.0015) | 0.001 |
| NT-proBNP(pg/ml ) | 1.0001 (1,1.0002) | 0.046 |
| CRP (mg/L) | 0.9993 (0.9911,1.0075) | 0.858 |
| ALB (g/L) | 0.98 (0.95,1.02) | 0.384 |

Abbreviations: BMI, body mass index; NYHA, New York heart association; COPD,Chronic obstructive pulmonary disease,CKD chronic kidney disease,CCI, Charlson Comorbidity Index; GFR glomerular filtration rate,CK-MB Creatine kinase isoenzyme,NT-proBNP N-terminal pro-B-type natriuretic peptide,Hs-cTn,high-sensitivity cardiac troponin,K Potassium ion,ALB Albumin,AST glutamic oxaloacetic transaminase, GCS,Glasgow Coma Scale.

**Supplementary Table 4** Baseline characteristics of HF Patients Without comorbidities

| Variables | Total  (n = 302) | Home  (n = 229) | Healthcare facility  (n = 73) | *P* |
| --- | --- | --- | --- | --- |
| Sex, male, n (%) | 105 (34.8) | 79 (34.5) | 26 (35.6) | 0.861 |
| Age(years),n (%) |  |  |  | 0.694 |
| <60 | 29 ( 9.6) | 23 (10) | 6 (8.2) |  |
| ≥60，＜80 | 169 (56.0) | 130 (56.8) | 39 (53.4) |  |
| ≥80 | 104 (34.4) | 76 (33.2) | 28 (38.4) |  |
| Occupation, n (%) |  |  |  | 0.003 |
| Farmer | 22 ( 7.5) | 21 (9.5) | 1 (1.4) |  |
| Urban Resident | 258 (87.8) | 187 (84.2) | 71 (98.6) |  |
| Worker/Others | 14 ( 4.8) | 14 (6.3) | 0 (0) |  |
| Admission way, n (%) |  |  |  | 0.001 |
| Non Emergency | 158 (52.3) | 132 (57.6) | 26 (35.6) |  |
| Emergency | 144 (47.7) | 97 (42.4) | 47 (64.4) |  |
| BMI (kg/m^2^) | 21.2 ± 4.2 | 21.3 ± 4.3 | 21.0 ± 3.7 | 0.607 |
| NYHA n (%) |  |  |  | 0.135 |
| II | 41 (13.6) | 36 (15.7) | 5 (6.8) |  |
| III | 168 (55.6) | 126 (55) | 42 (57.5) |  |
| IV | 93 (30.8) | 67 (29.3) | 26 (35.6) |  |
| Killip grade, n (%) |  |  |  | 0.693 |
| I | 93 (30.8) | 74 (32.3) | 19 (26) |  |
| II | 149 (49.3) | 112 (48.9) | 37 (50.7) |  |
| III | 49 (16.2) | 35 (15.3) | 14 (19.2) |  |
| IV | 11 ( 3.6) | 8 (3.5) | 3 (4.1) |  |
| **Laboratory values** |  |  |  |  |
| GFR（mL/min /1.73m^2^） | 73.5 ± 36.0 | 74.4 ± 37.8 | 70.6 ± 29.3 | 0.451 |
| Cre(mmol/L) | 82.5 (63.0, 107.5) | 80.9 (62.7, 107.1) | 84.3 (66.2, 108.9) | 0.666 |
| White blood cell(10^9/L) | 6.9 ± 3.1 | 7.1 ± 3.2 | 6.3 ± 2.8 | 0.07 |
| Red blood cell(10^9/L) | 3.8 ± 0.8 | 3.8 ± 0.8 | 3.9 ± 0.7 | 0.532 |
| Platelet(10^9/L) | 141.4 ± 62.1 | 139.7 ± 57.7 | 147.0 ± 74.8 | 0.39 |
| K(mmol/L) | 3.9 ± 0.7 | 3.9 ± 0.7 | 4.0 ± 0.7 | 0.54 |
| Na(mmol/L) | 138.3 ± 4.9 | 138.4 ± 5.1 | 138.1 ± 4.4 | 0.667 |
| D-dime(mg/L) | 1.1 (0.7, 1.8) | 1.1 (0.8, 1.9) | 1.1 (0.7, 1.4) | 0.204 |
| Hs-cTn(ng/mL) | 0.0 (0.0, 0.1) | 0.0 (0.0, 0.1) | 0.0 (0.0, 0.1) | 0.548 |
| AST( IU/L ) | 27.0 (20.0, 38.5) | 26.0 (20.0, 39.0) | 28.0 (20.2, 37.0) | 0.712 |
| CK-MB(IU/L) | 16.4 (12.2, 22.5) | 16.1 (12.2, 22.0) | 17.1 (12.4, 23.7) | 0.493 |
| NT-proBNP(pg/ml ) | 744.2 (266.7, 1633.7) | 726.8 (285.0, 1685.9) | 788.8 (232.8, 1384.4) | 0.453 |
| ALB(g/L) | 37.5 ± 5.0 | 37.4 ± 5.1 | 38.0 ± 4.3 | 0.411 |
| **Scoring system** |  |  |  |  |
| CCI.Score | 1.0 ± 0.4 | 1.0 ± 0.4 | 1.0 ± 0.3 | 0.814 |
| Movement | 6.0 ± 0.4 | 5.9 ± 0.5 | 6.0 ± 0.2 | 0.665 |
| DischargeDay(d) | 8.0 (6.0, 10.0) | 8.0 (6.0, 10.0) | 8.0 (7.0, 11.0) | 0.236 |
| **Outcome** |  |  |  |  |
| Readmission within 28-d, n (%) | 44 (14.6) | 29 (12.7) | 15 (20.5) | 0.096 |
| Readmission within 3 m, n (%) | 167 (55.3) | 121 (52.8) | 46 (63) | 0.128 |
| Readmission within 6m, n (%) | 261 (86.4) | 192 (83.8) | 69 (94.5) | 0.02 |

Categorical data are presented as percentages, continuous data are presented as median and interquartile range (IQR).Abbreviations: BMI, body mass index; NYHA, New York heart association; COPD,Chronic obstructive pulmonary disease,CKD chronic kidney disease,CCI, Charlson Comorbidity Index; GFR glomerular filtration rate,CK-MB Creatine kinase isoenzyme,NT-proBNP N-terminal pro-B-type natriuretic peptide,Hs-cTn,high-sensitivity cardiac troponin,K Potassium ion,ALB Albumin,AST glutamic oxaloacetic transaminase, GCS,Glasgow Coma Scale.

**Supplementary Table 5**  Association between discharge destination and readmission in multiple regression model.

|  | **Variable** | **Unadjusted model** | |  | **Model 1** | |
| --- | --- | --- | --- | --- | --- | --- |
|  |  | HR (95%CI) | *P* value |  | HR (95%CI) | *P* value |
| Readmission within 28-d | Home | 1(Ref) |  |  | 1(Ref) |  |
|  | Healthcare Facility | 1.72 (0.92~3.22) | 0.087 |  | 2.1 (0.97~4.54) | 0.061 |
| Readmission within 3m | Home | 1(Ref) |  |  | 1(Ref) |  |
|  | Healthcare Facility | 1.36 (0.97~1.9) | 0.079 |  | 1.6 (1.03~2.5) | 0.037 |
| Readmission within 6m | Home | 1(Ref) |  |  | 1(Ref) |  |
|  | Healthcare Facility | 1.29 (0.98~1.7) | 0.072 |  | 1.45 (1.01~2.09) | 0.043 |

Model 1:was adjusted for sex,age,BMI;NYHA, Killip grade,type of heart failure, white blood cell, platelet, cre, D-dimer, Hs-cTn, NT-pro BNP, ALB,discharge ,Occupation status and Admission way.


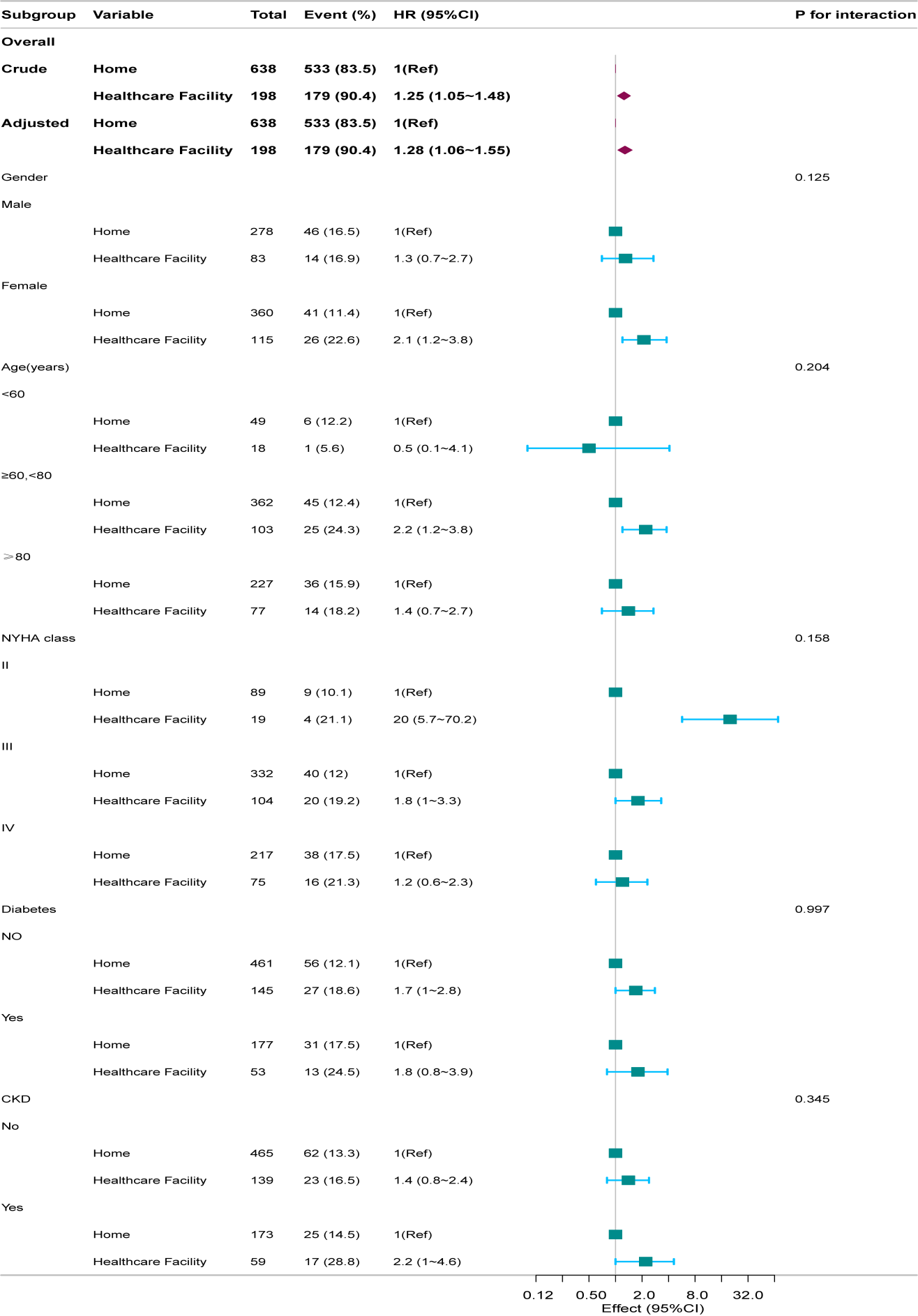


**Supplementary Fig 1.** Association between discharge destination and the hazard ratio of readmission within 28-days according to subgroup.


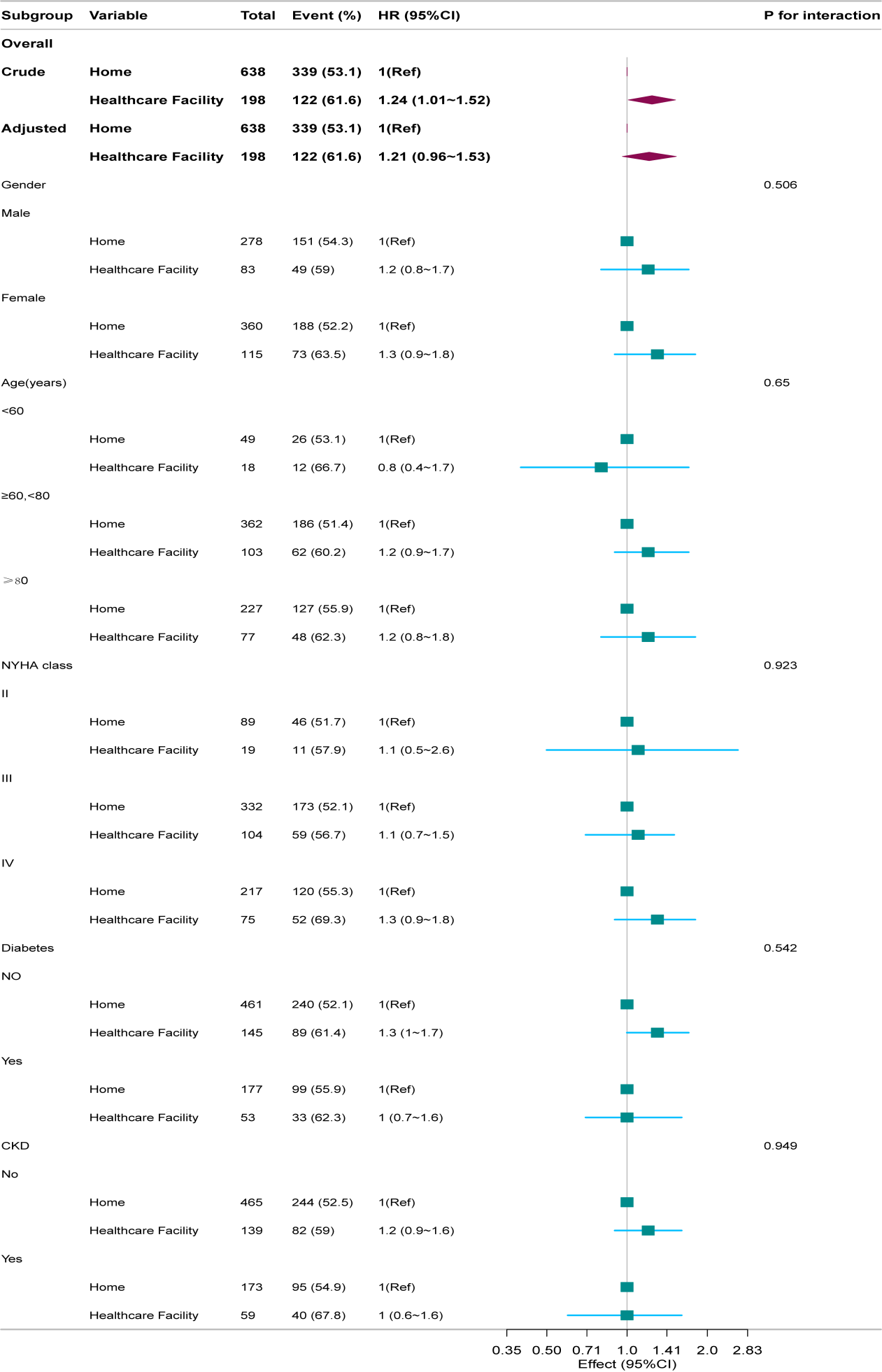


**Supplementary Fig 2.** Association between discharge destination and the hazard ratio of readmission within 3 months according to subgroup.
